# Colorectal cysts as a validating tool for CAR therapy

**DOI:** 10.1186/s12896-020-00623-0

**Published:** 2020-06-01

**Authors:** Pierre Dillard, Maren Lie, Elizabeth Baken, Viola Hélène Lobert, Emmanuelle Benard, Hakan Köksal, Else Marit Inderberg, Sébastien Wälchli

**Affiliations:** 1 Department of Cellular Therapy, Department of Oncology, Oslo University Hospital-Radiumhospitalet, Oslo, Norway; 2 Department of Molecular Cell Biology, Institute for Cancer Research, Oslo University Hospital-Radiumhospitalet, Oslo, Norway

**Keywords:** Immunotherapy, CAR T cells, Spheroids, Microscopy

## Abstract

**Background:**

Treatment of cancers has largely benefited from the development of immunotherapy. In particular, Chimeric Antigen Receptor (CAR) redirected T cells have demonstrated impressive efficacy against B-cell malignancies and continuous efforts are made to adapt this new therapy to solid tumors, where the immunosuppressive tumor microenvironment is a barrier for delivery. CAR T-cell validation relies on in vitro functional assays using monolayer or suspension cells and in vivo xenograft models in immunodeficient animals. However, the efficacy of CAR therapies remains difficult to predict with these systems, in particular when challenged against 3D organized solid tumors with highly intricate microenvironment. An increasing number of reports have now included an additional step in the development process in which redirected T cells are tested against tumor spheres.

**Results:**

Here, we report a method to produce 3D structures, or cysts, out of a colorectal cancer cell line, Caco-2, which has the ability to form polarized spheroids as a validation tool for adoptive cell therapy in general. We used CD19CAR T cells to explore this method and we show that it can be adapted to various platforms including high resolution microscopy, bioluminescence assays and high-throughput live cell imaging systems.

**Conclusion:**

We developed an affordable, reliable and practical method to produce cysts to validate therapeutic CAR T cells. The integration of this additional layer between in vitro and in vivo studies could be an important tool in the pre-clinical workflow of cell-based immunotherapy.

## Background

Adoptive cell transfer (ACT) is the transfer of patient’s immune effector cells (T- or NK- cells) to obtain anti-tumor activity. This strategy represents new hope in the treatment of cancer. These cells can be genetically modified with a receptor that will guide them to their target, the tumor cells, and destroy them. The most spectacular example of such therapy has been the use of a Chimeric Antigen Receptor (CAR) directed against the B-cell marker CD19 [1, 2]. CAR consists of specific antibody fragments, the antigen binding domain designed as single chain variable fragment (scFv), linked to the T-cell signaling domains. Although there are several designs, the most commonly used versions referred to as second-generation CAR designs, consist of CD3ζ for TCR signaling and one co-stimulatory domain (CD28, 4-1BB, OX40, etc.) [3, 4]. The development of efficient CAR therapies targeting CD19 B-cell malignancies [5, 6] represented a paradigm shift that re-oriented the cell-based immunotherapy field towards this new type of treatment. CAR T cells have shown to be able to efficiently control haematological malignancies, “liquid tumors”, whereas “solid tumors” revealed to be more challenging. This can be attributed to several factors, but two main reasons seem to have emerged: the low number of tumor-specific surface antigens and the immunosuppressive tumor microenvironment [7].

Additionally, most current in vitro validation strategies rely on two-dimensional (2D) systems which do not properly address solid tumor challenges. Classically, 2D in vitro systems involve a mixture of CAR T cells and target cancer cell lines as monolayers to assess the functionality and specificity of these effector cells. Although these strategies are important and vital steps of the pre-clinical development, they do not take complex morphology and three-dimensional (3D) organization of the cancer cells into consideration [8]. Cancer cells cultured in 3D systems, referred to as spheroids, acquire new phenotypic traits through changes in gene expression profile [9] which might influence the recognition by redirected effector cells. These can be modifications in regulatory receptor expression, as well as physical constraints on the actin cytoskeleton, which will result in changing the T-cell recognition mechanism of the cognate target [10, 11]. In this aspect, the physical constraints mediated by actin to form these cysts will directly impact the ligand friction, hence the T-cell recognition. Birgersdotter and colleagues demonstrated that a Hodgkin lymphoma (HL) cell line only acquires a gene expression profile that is similar to primary tumor samples if grown in 3D [12]. Such alteration in gene expression was observed in various tumor model and numerous reports pointed that cells grown in 3D culture are more similar to their tissue of origin than their 2D counterpart [13–17]. In this aspect, spheroids offer more relevant in vitro models as opposed to standard 2D systems. Another advantage of such systems is the prediction of in vivo studies which are seen as the final step in the validation process of a given CAR. Indeed, it has been proposed that spheroids present numerous similarities with in vivo models [17–19] and are valuable tools to assess CAR efficacy more critically [20].

Recent studies have led to the development of organoids, regarded as complexified spheroids [21]. Indeed, they are derived from stem cells, possess multiple cell lineages, recapitulate organ physiological parameters and can be maintained for extended periods [22, 23]. Unfortunately, organoids and especially patient-derived ones represent a technique that is facing many challenges such as the supply of patient material, heterogeneity, reproducibility, time, and cost. Their implementation in the development process of a therapy would be valuable but remains difficult.

Self-organization of the colorectal adenocarcinoma cell line Caco-2 into cysts was described by Jaffe et al. [24] They showed that these cells have the capacity to grow in a three-dimensional (3D) matrix, generate a cyst-like structure, where the apical surface of each epithelial cell faces a fluid-filled central lumen. The formation of such hollow lumens is regarded as a key difference between spheroids and cysts [25]. We report here a simple and affordable method to develop cysts, which are minimal organoids, but presenting a higher level of 3D complexity over a simple spheroid. Indeed, despite being made from one cell type, enterocytes, cysts present an oriented 3D structure. In order to test these cysts with clinically validated CAR redirected T cells, we transduced Caco-2 cells with *CD19* gene. We first showed that CD19CAR T cells were able to kill these cells either as a 2D monolayer or as cysts. We further demonstrated the adaptability of our method to various techniques: super-resolution microscopy, high-throughput live imaging and bioluminescence (BLI) assays. Such flexibility permitted a complete characterization of the cyst structure as well as a quantitative and qualitative description of CD19CAR T-cell cytotoxicity and ability to extravasate through complex matrices. This protocol represents a step between classical spheroids and more complex organoids while being scalable, inexpensive, reliable and easy to adapt to various environments and quantifications methods.

## Results

**Fig. 1:**
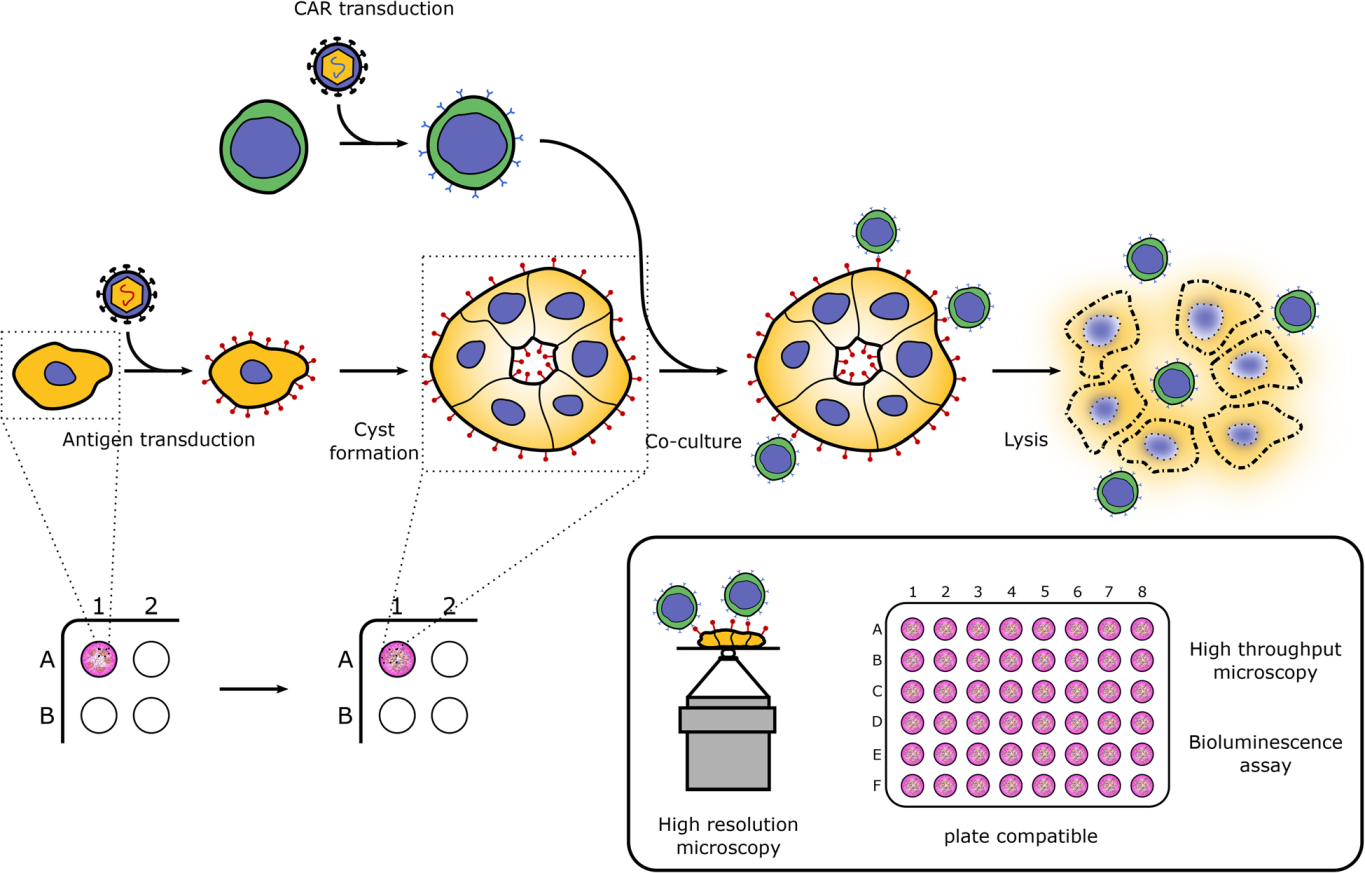
Protocol principle

As described above, the principle of our technique relies on the formation of cysts from stably transduced Caco-2 cells as a tool to validate CAR T-cells efficacy and mobility (Fig. 1).

**Fig. 2:**
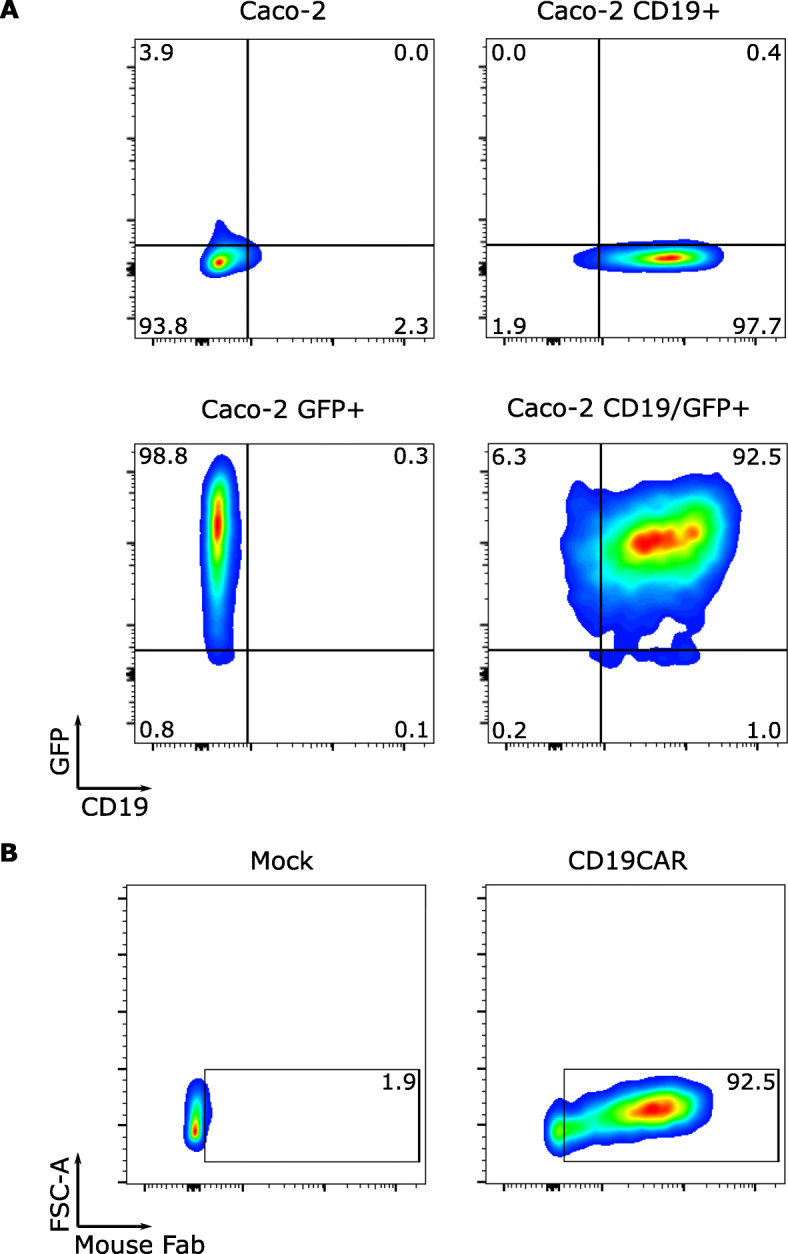
Retroviral transduction of Caco-2 and T cells. **a** Representative FACS flow showing Caco-2 cells retrovirally transduced to express GFP, CD19 or both. **b** Representative FACS flow showing T cells retrovirally transduced to express the CD19CAR construct

We first established a cell line from the human colorectal Caco-2 stably expressing the antigen of interest, CD19, with or without a GFP-luciferase construct to be used for BLI killing assay (see below and [26, 27]). Cells were transduced using gammaretrovirus and sorted by FACS in order to obtain a pure population with high expression of both transgenes (Fig. 2a). The effector T cells were transduced with a CD19CAR construct [26] and the expression levels of the construct was analyzed by flow cytometry (Fig. 2b).

**Fig. 3:**
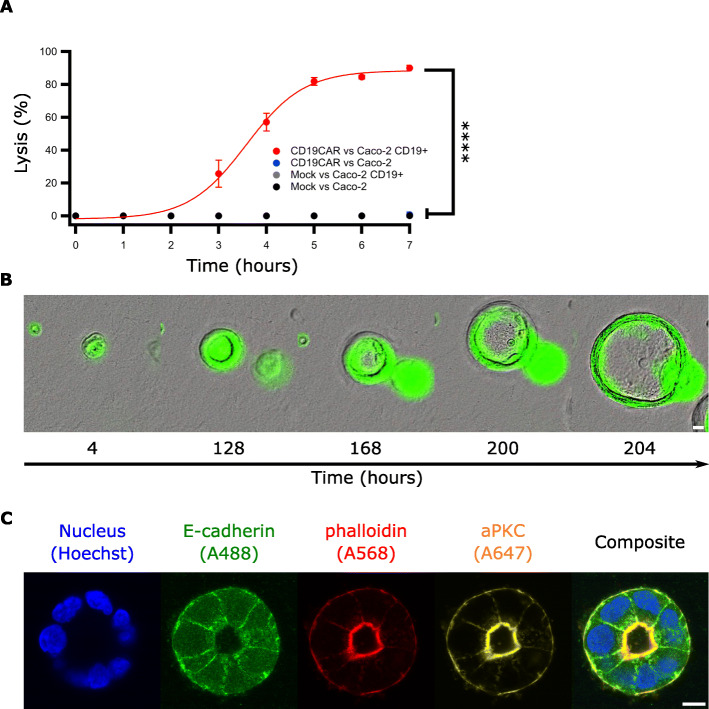
CD19 is expressed on the surface of Caco-2 cells and do not hinder their ability to form cysts. **a** BLI killing assay of Caco-2 cells expressing CD19 or not, co-cultured with CD19CAR or Mock T cells (E:T ratio of 1:10). Data represent mean ± S.D. of hexaplicates. Representative data from one of three experiments are shown. Statistics analysis were conducted from timepoints 3 to 7 (2-way ANOVA). **b** Time lapse of Caco-2 GFP+/CD19+ cysts formation observed with a high-throughput microscope. Scale bar represents 20 μm. **c** Airyscan micrographs showing the establishment of a basal/apical polarity on a Caco-2 organoid marked by Hoechst 33342, PKC-ζ, E-cadherin and actin. Scale bar represents 10 μm

Next, we verified that this cell line could be recognized and killed by CD19CAR T cells using BLI assay. As shown, the cytotoxic activity of the CD19CAR T cells was specific and restricted to Caco-2 CD19+ cells since CD19- Caco-2 were not killed. As a control, we also used mock T cells which did not react with any of the targets (Fig. 3a and Additional file 1A). This assay demonstrates that CD19 antigen was correctly presented and folded on the surface of the Caco-2 cells and that CD19CAR T cells could access and recognize the target.

We, then, tested whether CD19 expression affected the development of a 3D cyst. The formation of the lumen (Fig. 3b), as well as the apical- basal orientation of the cells, proved to be similar to what has been observed in wild type Caco-2 cells. More specifically, we observed enhanced actin accumulation at internal surfaces facing a central lumen with distinct adherens junctions (visualized by epithelial cadherin [E-cadherin] staining) and atypical PKC (aPKC), an apical membrane marker, localizing close to the tight junctions facing the luminal space (Fig. 3c).

**Fig. 4:**
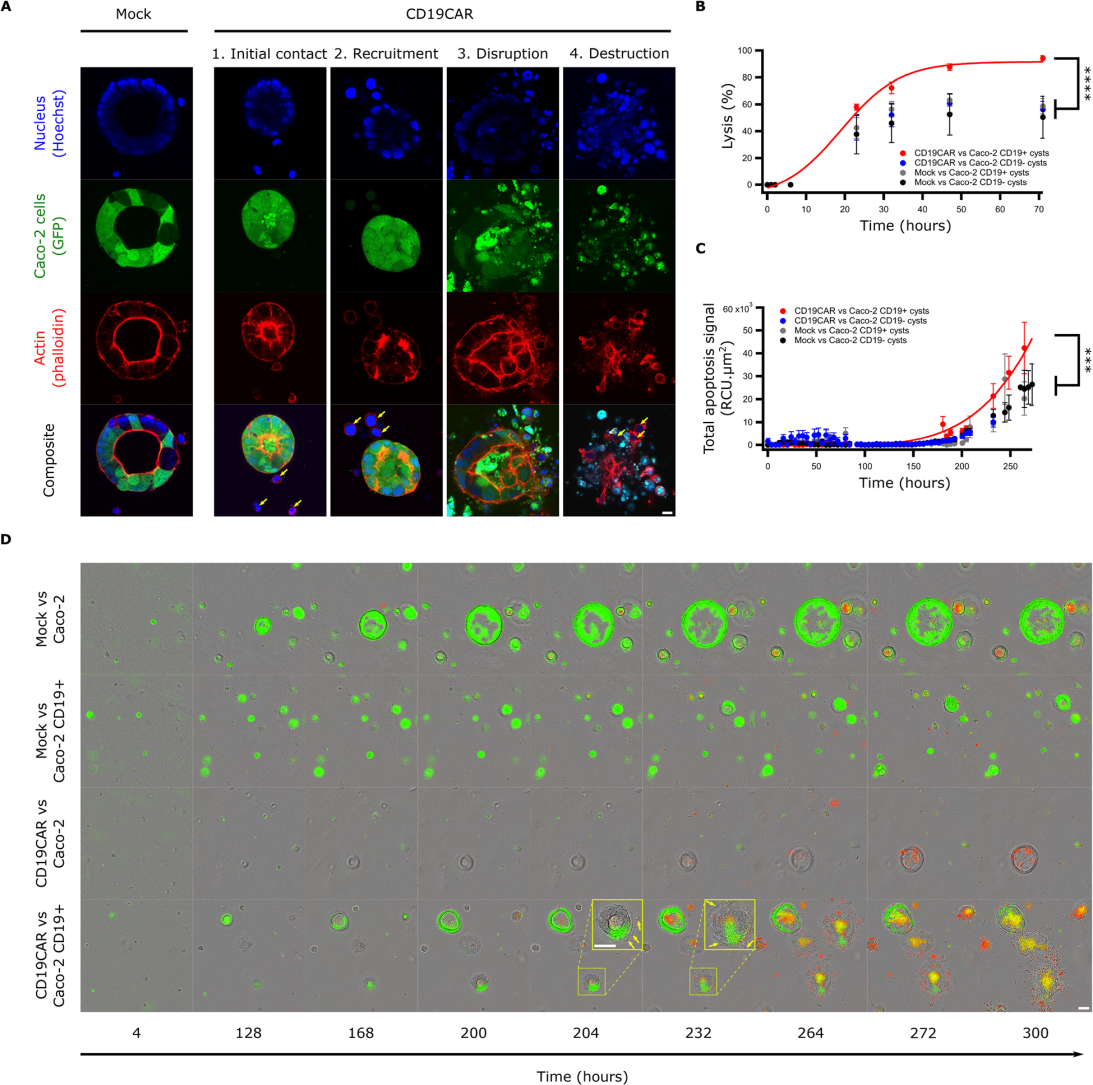
CD19CAR T cells demonstrate killing of Caco-2 CD19+ cysts. **a** Representative confocal micrographs showing specific killing of Caco-2 GFP+/CD19+ cysts mediated by CD19CAR T cells. Cells were marked with Hoechst 33242 and actin. Scale bar represents 10 μm. Yellow arrows outline the presence of T-cells. **b** BLI killing assay of Caco-2 cysts expressing CD19 or not, co-cultured with CD19CAR or Mock T cells. Data represent mean ± S.D. of hexaplicates. Representative data from one of three experiments are shown. Statistics analysis were conducted from timepoints 3 to 7 (2-way ANOVA). **c** Total apoptosis signal as measured by Annexin V of Caco-2 cysts expressing CD19 or not, co-cultured with CD19CAR or Mock T cells and imaged by high throughput microscope. Data represent mean ± S.D. of octuplicates. Representative data from one of three experiments are shown. Statistics analysis were conducted at timepoint 232 (1-way ANOVA). **d** Corresponding time lapse of the killing assay described in C. Scale bars represent 80 μm. Yellow arrows outline the presence of T-cells

Killing efficacy of CD19CAR T cells against Caco-2 cysts was assessed through several means to demonstrate the versatility of this technique and its adaptability to various pre-clinical validation processes. After 6 days of culture, matrix-embedded cysts were co-incubated with CD19CAR or Mock T cells deposited on top of the Matrigel-collagen mixture. Confocal imaging revealed that CD19CAR T cells were able to extravasate through the matrix to reach the cysts. Upon initial recognition, more T cells will be recruited and the cyst will be ultimately destroyed (Fig. 4a and Additional file 1B). Despite the ability to bring important qualitative measurements, high resolution confocal microscopy is not ideal to obtain quantitative metrics. We, therefore, adapted the classical BLI assay to the cysts and were able to observe that CAR T cells could reach the cysts and kill them specifically (Fig. 4b). Finally, we used a high-throughput live imaging method (Incucyte S3) and confirmed previous observations made with confocal microscopy and BLI assay. Here, CD19CAR T cells needed around 24 h to extravasate through the matrix and reach the cysts. This phase was followed by subsequent aggregation of more CD19CAR T cells and led to the disruption of the structure and ultimately to the killing of the surrounding cysts (Fig. 4d). Quantification of the apoptosis signal confirmed the specificity and efficiency of the CD19CAR T cells (Fig. 4c).

## Discussion

The use of spheroids as an innovative tool to validate future cancer treatment has become a field of growing interest in the past years. The method further holds a lot of promise in terms of tumor microenvironment mimicking as well as gene profiling [9]. However, spheroids represent solely aggregates of cells, from a single line, without complex spatial organizations, therefore, do not represent the full complexity of an organ [25]. On the other hand, organoids despite their advantages, are costly, heterogenous and long to produce making it challenging to integrate them in a validation process. In this respect, organoids cannot be viewed as a cheaper alternative to in vivo experiments. The method presented in this publication was adapted from Jaffe et al. [24] to the CAR T-cell development using cysts which can be considered as sophisticated spheroids. Due to its versatility, it can be adapted easily to other tumor cell lines that have been shown to form similar 3D structures [28, 29]. It conserved the advantages of spheroids (inexpensive, reliable and easy to set-up) with an oriented heterogenetic 3D structure more accurately mimicking a complex cellular organization. Furthermore, gene expression of Caco-2 cells has been shown to change upon transitioning from a 2D culture to a 3D cyst organization [30]. More generally, several reports have pointed that gene expression of cells grown in 3D culture are more similar to their tissue of origin than their 2D counterpart [13–17, 31]. We coupled this method to several validation tools such as BLI assays and high-throughput imaging and demonstrated that it could be used to monitor the ability of CAR T cells to destabilize a tumor structure. In order to reduce the variations relative to antigen distribution or density which could also impact the killing efficiency, we have normalized our system by using a common CAR target, CD19, and a validated CAR construct (fmc63). However, we have generated preliminary data indicating that endogenous antigens can also be targeted (our unpublished data). The direct visualization as well as the generation of quantitative metrics brought by our protocol are still relevant independent of the target background and remain valuable assets in the pre-clinical development of any anti-cancerous therapy.

This method is so far only limited to a certain type of adherent cell line (here Caco-2) and, as previously mentioned, additional effort should be made to extend this protocol to other model cell lines. Moreover, regarding the high-throughput live imaging adaptability, various instruments are now available and should be evaluated.

## Conclusions

We report here an affordable and simple method to use cysts for testing the efficacy of CAR T cells. We demonstrated that it could be used in different experimental setups and generate microscopic data to understand the killing process of T cells, making it a powerful tool in the context of anti-cancer treatment validation.

## Methods

### Cell lines, media and reagents

Colorectal cancer cell line Caco-2 was obtained from ATCC (catalogue number: HTB-37, USA). Cell line was tested for mycoplasma contamination using a PCR based detection kit (Venor®GeM, Minerva Biolabs). Caco-2 cells were cultured in DMEM (Gibco, Thermo Fisher Scientific, USA) supplemented with 15% Fetal calf serum (FCS) (Gibco, Thermo Fisher Scientific, USA).

The study was approved by the Regional Committee for Medical Research Ethics (Oslo, Norway) (REC approval no: 2013/624, 2016/2247). T cells from healthy donors were expanded using a protocol adapted for pre-clinical production of T cells employing CD3/CD28 Dynabeads essentially as previously described [32]. In brief, PBMCs were isolated from Buffy coats by density gradient centrifugation and cultured with Dynabeads (CTS™ Dynabeads™ CD3/CD28, provided by Gibco, Life Technologies AS, Norway) at a 3:1 ratio in complete X-Vivo 15 medium with 100 U/mL IL-2 (Proleukin, Novartis Healthcare, USA) for 10 days. After 10 days’ expansion, CD3/CD28 Dynabeads were removed and T cells were frozen or used directly.

### Retroviral particle production

Viral particles were produced as described in [33] and used to transduce T cells and Caco-2 cells. In brief, 1.2.10^6^ Phoenix-AMPHO (ATCC, Catalogue number: CRL-3213) cells were plated. Transfection was performed using Extreme-gene 9 (Roche) with a mix of DNA including the retroviral packaging vectors and the expression vector to an equimolar ratio. After 24 h, the medium was replaced with 1% HyClone FCS-containing DMEM and the cells were transferred to a 32 °C incubator. Supernatants were harvested after 24 h and 48 h of incubation.

### Transduction of cells

PBMCs were incubated for 2 days in a 24 wells-plate coated with CD3 and CD28 at 1.10^6^ cells/mL. A 24-well plate was coated with 50 μg/mL of retronectin (Takara, Shiga, Japan) during 3 h at room temperature before being washed with PBS and blocked with a solution of 1 mg/mL of FBS during 30 min. One milliliter of virus solution was deposited in each well and topped with 500 μL of activated T cells or Caco-2 cells at a concentration of 0.3.10^5^ cells/mL. The plate was then incubated for 30 min at 37 °C, 5% CO2 for 30 min, sealed and then spun down at 750 g, 32 °C during 60 min before being placed back in the incubator. The same spinoculation step was repeated the following day before the cells being collected, spun down, washed and resuspended in complete X-Vivo 15 medium (for CD19CAR cells) or complete DMEM medium (for Caco-2 cells) for 2 days before the expression of the construct was checked and the cells expanded using the procedure described above.

### Antibodies and flow cytometry

CAR expression was detected by anti-mouse Fab antibody (Jackson ImmunoResearch, West Grove, PA, USA). CD19 expression was detected by anti-human CD19 APC (Invitrogen, Thermo Fisher Scientific, Germany). Cells were acquired on a BD FACSCanto flow cytometer and the data analyzed using FlowJo software (Treestar Inc., Ashland, OR, USA).

### Bioluminescence-based cytotoxicity assay

Luciferase-expressing Caco-2 cells were counted and resuspended at a concentration of 3.10^5^ cells/mL. Cells were given D-Luciferin (75 μg/ml; Perkin Elmer) and were placed in 96-well white round-bottomed plates as 100 μl cells/well. CAR T cells were added at a 10:1 E:T ratio. In order to determine spontaneous and maximal killing, wells with target cells only or with target cells in 1% Triton™ X-100 (Sigma-Aldrich) were seeded. Cells were left at 37 °C and the bioluminescence was measured with a luminometer (VICTOR Multilabel Plate Reader) as relative light units (RLU) at indicated time points. Target cells incubated without any effector cells were used to determine baseline spontaneous death RLU at each time point. Triplicate wells were averaged and lysis percentage was calculated using the following equation: % specific lysis = 100x (spontaneous cell death RLU- sample RLU)/(spontaneous death RLU – maximal killing RLU). Sigmoid curves (no Hill equation) were fitted for every set of points (using Igor Pro 8.1, Wavemetrics, USA) as guide for the eye with standard deviation as the weighting factor.

### 3D structure formation

Caco-2 cells (GFP+ with or without CD19) were trypsinized and resuspended as a single cell suspension at a concentration of 1.10^6^ cells/mL. For 1 mL of cysts mixture, 400 μL of Caco-2 cells were mixed with 400 μL of matrigel (BD Bioscience) and 200 μL of Rat collagen I (final concentration of 1 mg/mL, Cultrex). One hundred microliters of the mixture was dropped per well either into 96 wells plate for BLI assays and high throughput imaging or in Ibidi u-Slide 8 well treated with ibiTreat #1.5 polymer (Ibidi) before being covered with 100 μL of complete DMEM. Plates for live imaging were incubated into an Incucyte S3 (Essen Biosciences, UK) and imaged every 4 h. In all case, cysts were let to grow for 6 days prior to use.

For BLI assay, cysts were let to grow in 96-well white round-bottomed plates. At day 6, cysts were incubated with 100 μg/ml of D-Luciferin for 16 h. T cells were then added on top of the collagen-matrigel mixture by adding 5.10^5^ cells/well (roughly at a 1:10 E:T ratio) in 50 μL of complete DMEM medium. Killing was assessed in a similar way as described above.

For high throughput imaging assay, at day 6, 50 μL of a 1:200 solution of Annexin V red (Essen Biosciences, UK) diluted in complete RPMI 1640 was added per well and the plate was subsequently incubated at 37 °C, 5%CO2 for 15 min. Mock and CD19CAR T cells previously washed and resuspended in complete DMEM medium were introduced in each well at a final concentration of 5.10^5^ cells/well. The plate was then put into an Incucyte S3 with the same settings as described above. Analysis of cytotoxicity was performed using Incucyte software. Metrics were then extracted and corrected using Igor Pro 8.1 (Wavemetrics, USA).

For confocal imaging, at day 4, 2.10^4^ Mock or CD19CAR T cells were placed on top of each well. Then at day 6, cysts were fixed in 4% formaldehyde (Sigma-Aldrich) for 30 min. After PBS rinsing, unreacted aldehyde groups were quenched with 50 mM of NH_4_Cl (Sigma-Aldrich) for 20 min. Cysts were permeabilized with 0.5% Triton X-100 for 15 min before being incubated with aPKC zeta (C-20) (Santa Cruz) and E-cadherin (32A8) (Cell Signaling) overnight at 4 °C in 0.05% of saponin in PBS. Cysts were then washed three times with 0.05% of saponin in PBS before being marked with secondary antibodies Alexa488 donkey-anti-mouse (Jackson ImmunoResearch) and Alexa568-donkey-anti-rabbit (Molecular Probes). Slides were then washed extensively with PBS before nuclei being marked with 20 μM of Hoechst 33352 for 5 min. After a final PBS wash, cysts were imaged with a Zeiss LSM 880 Airyscan with a 1.4 NA water immersion 40x objective.

### Statistical analysis

Statistics were made with 1-way ANOVA with Brown-Forsythe and Welch and Dunett T3 corrections (single timepoint comparison) or 2-way ANOVA with Greenhouse-Geisser and Tukey corrections (multiple timepoints comparison). * *p* < 0.05, ** *p* < 0.01, *** *p* < 0.001, **** *p* < 0.0001. All statistical analyses were performed using R.

## Supplementary information

**Additional file 1.** Independent BLI assays of Caco-2 CD19+ (as single cells or as cysts) co-incubated with CD19CAR T-cells. Three independent repetitions of the experiments displayed in Figs. 3a and 4b.

## Data Availability

The datasets used and analyzed during the current study are available from the corresponding author on reasonable request.

## Abbreviations

2D: 2 dimensional
3D: 3 dimensional
ACT: Adoptive cell transfer
BLI: Bioluminescence
CAR: Chimeric Antigen Receptor
FCS: Fetal calf serum
HL: Hodgkin lymphoma
scFv: Single chain variable fragment
