# Supplementary material for: Colorectal cysts as a validating tool for CAR therapy
Source: BMC Biotechnol. 2020 Jun 1;20:30. doi: 10.1186/s12896-020-00623-0 (PMC7268759; doi:10.1186/s12896-020-00623-0)
Supplement: Supplementary file 1 — Additional file 1. Independent BLI assays of Caco-2 CD19+ (as single cells or as cysts) co-incubated with CD19CAR T-cells. Three independent repetitions of the experiments displayed in Figs. 3a and 4b. [file 12896_2020_623_MOESM1_ESM.pdf]

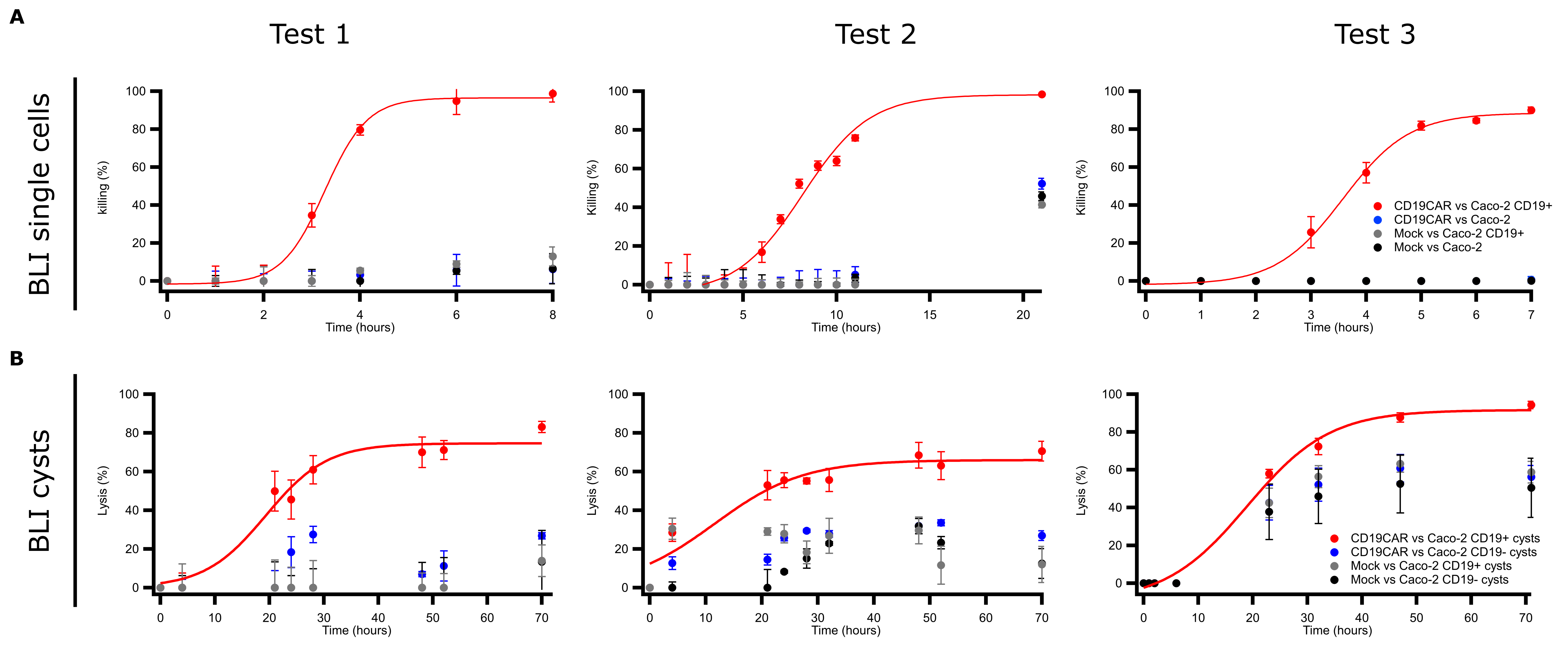


Additional file 1:

A. Three independent BLI killing assays of Caco-2 cells expressing CD19 or not, co-cultured with CD19CAR or Mock T cells (E:T ratio of 1:10). Data represent mean ± S.D.

B. Three independent BLI killing assays of Caco-2 cysts expressing CD19 or not, co-cultured with CD19CAR or Mock T cells. Data represent mean ± S.D.
